# The effect of a career choice guidance on self-reported psychological problems

**DOI:** 10.3389/fpsyg.2014.00547

**Published:** 2014-06-04

**Authors:** E. S. Kunnen

**Affiliations:** Department of Developmental Psychology, University of GroningenGroningen, Netherlands

**Keywords:** career choice problems, psychological problems, intervention, identity development, late adolescents

## Abstract

Late adolescents with career choice problems often have psychological problems as well. The starting point of this study was the question of career choice counselors whether potential clients with career choice problems and psychological problems could be accepted in career choice intervention, or whether it was better to advise them to seek help for their psychological problems. We investigated whether a successful career choice intervention reduced psychological problems, and whether this program was equally effective in participants with low and with high levels of psychological problems. Participants were 45 Dutch students (age 17–24) with career choice problems. They had above average levels of self-reported psychological problems before the start of the intervention. These problems decreased significantly following the intervention. With regard to vocational commitment development, the intervention was equally effective for participants with low or average and with (very) high levels of psychological problems before the start of the intervention.

## INTRODUCTION

The starting point for this study was the question of career choice counselors whether late adolescents with career choice problems and with high levels of psychological problems – such as anxiety or depression – could be included in their career choice interventions, or whether it is better to address their psychological problems before addressing their career choice problems. In this study we aim to shed some light on that question by investigating whether the level of psychological problems reduces after a career choice intervention, and whether a career choice intervention is equally effective in participants with low and with high levels of psychological problems.

## CAREER CHOICE DEVELOPMENT FROM THE PERSPECTIVE OF IDENTITY THEORY

Making satisfying career choices can be seen as the development of strong and adaptive vocational commitments, and thus as an important aspect of identity development (Kroger, 2007). It is also a difficult task: many late adolescents struggle with it (Feldman, 2003; Gati and Asulin-Peretz, 2011). Career choice problems can be seen as a manifestation of problematic identity development (Bosma and Kunnen, 2001; Luyckx et al., 2008; Kroger et al., 2010), and more specifically, as problems in commitment formation in the domain of vocational identity. Exploration and commitment formation are seen as two crucial dimensions in identity development (Marcia, 1966). By exploration we mean that individuals are seriously considering different possibilities before they form commitments. Commitment formation refers to the process of making choices in which individuals are personally involved, thus choices about issues that really matters to them. Optimally, identity development proceeds from an initial state in which there are either no commitments or commitments that are adopted from significant others [Marcia (1966) coined these as diffused and foreclosed statuses, respectively] via a period characterized by the absence of commitments and high exploratory activity (the moratorium status) to the identity achieved status. In this status the individual has developed mature commitments, that is, had made strong and satisfying choices (such as career choices) that fit in with the own values, interests and abilities and with the possibilities offered by the environment (Marcia, 1966; Kunnen et al., 2008; Kroger et al., 2010). Commitments that have been developed on the basis of a period of exploration are flexible, strong, and give the person a sense of direction and of knowing who one is (Bosma and Kunnen, 2001; Marcia, 2002). Research suggests that having commitments is important for ones well-being (Vleioras and Bosma, 2005; Schwartz et al., 2011). Applied to the domain of career choice, making satisfying and adaptive vocational choices can be seen as the development of achieved commitments in the vocational domain. However, this does not mean that career choice development is restricted to the vocational domain only. A career choice is satisfying and adaptive if the choice represents a match between characteristics of the person and of the study or career, and if the person can integrate his or her choice in his overall identity. This implies that also personal and global commitment development are relevant aspects in career choice development. Therefore, we will use vocational, personal and global commitment development as indicators of career choice development.

## CAREER DEVELOPMENT IN THE NETHERLANDS

The development of career choice commitments is considered an important developmental task, and it is seen as one of the most important parts of identity development in late adolescence in the western world (Bosma, 1985; van Hoof, 1997). However, especially in the Netherlands career choice development is considered very important, because failure in making the right vocational choice has major consequences. Higher education in the Netherlands is characterized by a large amount of specific different studies and new students have to choose for one of these studies, such as for example psychology, or economy, or sociology. Figures for drop out in higher education show that in the Netherlands about 30% of all students stop with the study in the first year, mostly because the students feel they have made the wrong choice (Trendrapport, 2010). Drop-out often means that the students have to wait until the next year before they can start a new study. Moreover, the governmental support for students is restricted in time, so dropout costs the student time and money. Thus, developing satisfying vocational commitments is at the same time important and difficult for many late adolescents and career choice counselors report a rapid growth in the number of clients (personal communications). As a consequence, development and evaluation of career choice interventions is considered highly important. Although a vast amount of research demonstrates general effectiveness of career choice interventions, more knowledge is needed concerning their effectivity in different groups (Brown and Ryan Krane, 2000). Brown and Ryan Krane (2000) even state that career counseling is definitely found to be effective, and that we now need research that explores the potential moderating effects of clients’ sex, race, sexual orientation, etc. That means that we have to investigate what type of characteristics facilitate or hamper the effects of career choice intervention in different individuals. One of the potentially relevant characteristics is the presence of psychological problems. In the next section we will discuss literature concerning the relation between psychological problems and career choice problems.

## CAREER CHOICE AND PSYCHOLOGICAL PROBLEMS

Empirical evidence shows that late adolescents with career choice problems often have high levels of psychological problems. For example, Feldman (2003) found several factors, such as low levels of self-esteem and self-efficacy, and low cognitive abilities to be positively related to career indecision. University students who made a career decision were less depressed than students who were undecided (Rottinghaus et al., 2009). Also Creed et al. (2004) found positive relations between indicators of adaptation and wellbeing, and indications of career decisiveness: high levels of self-efficacy, optimism, self-esteem, and low levels of pessimism and a low tendency to perceive external barriers were significant predictors for career decision, in which self-esteem emerged as the sole significant individual predictor. In another study, Creed and Patton (2003) found that self-efficacy, age, career decidedness (certainty) and work commitment were the main predictors of career maturity attitude. Kunnen et al. (2009) found that individuals who sought help for their career choice problems had above average levels of psychological problems and less effective coping strategies. Skorikov (2007) suggests that because of the relation between career choice problems and psychological problems, and because career choice is so important for large groups of late adolescents, career choice counseling should be a standard component of general prevention programs for adolescents. Concluding, literature shows that students who do have career choice problems on average have higher levels of different kinds of psychological problems.

## THE DIRECTION OF THE RELATION BETWEEN CAREER CHOICE PROBLEMS AND PSYCHOLOGICAL PROBLEMS

However, most research into the relation between career choice problems and psychological problems is correlational in nature and thus does not help to answer the question of practitioners whether intervention should first address the psychological problems or the career choice problems (Rottinghaus et al., 2009). If psychological problems hamper the career choice process, intervention should be directed toward these psychological problems. But if career choice problems result in psychological problems, career choice intervention may result in a decrease of the psychological problems as well. Research into this causal relation is scarce and inconclusive. Creed et al. (2006) found no effects across time of self-efficacy on career choice indecision. The same authors concluded in another study (Creed et al., 2007) that self-esteem has a mediating but no direct effect on career related variables. On the other hand, Skorikov and Vondracek (2007) followed 14-year old adolescents during 1 year and found that a positive career orientation had a significant negative longitudinal effect on problem behavior. Skorikov (2007) found small but significant effects of career preparation on adjustment factors such as self-esteem, social adaptation, and emotional stability, but he found no effect of career preparation on depression and anxiety.

From the perspective of identity theory we may expect that psychological problems such as anxiety and depression may result from career choice problems. Persons who have no commitments in the vocational domain have a diffused or a moratorium status in this domain. These two statuses are related to a broad range of psychological complaints (Marcia, 1993). Especially in adolescence and emerging adulthood, people are confronted with both individual changes and new environmental demands that force them to make decisions concerning their future occupational life (Arnett, 2000, 2007). Skorikov (2007) suggests that progress in career preparation can have an effect on positive feelings of adjustment as a result of feelings of competence and a sense of accomplishment in the fulfillment of a major developmental task. Not being able to make satisfying career choices may be felt as a failure, in the eyes of the individual and also in the eyes of the outside world. Not being able to fulfill the most important developmental task and to meet the expectations of parents and others may result in low self-esteem and psychological problems. Skorikov (2007) found evidence for positive effects of career choice preparation on a broad array of indicators of adjustment, such as a reduction in depression and anxiety. Montgomery et al. (2008) state that identity development problems are at the basis of many problems and problems of late adolescents, and they stress that intervention in this age group should pay more attention to solving identity problems. As identity development runs from a diffused or foreclosed status via a period of exploration to the formation of achieved commitments, intervention that aims to affect identity development should focus on firstly the stimulation of exploration, and secondly, on the formation of commitments that are based on the experiences that emerge from this moratorium phase.

### SUMMARIZING:

In the literature no support is found for the assumption that psychological and behavioral functioning affects career choice development, but there is some evidence for effects of career choice development on psychological functioning. Theory and research suggest that career choice problems in late adolescents (conceptualized as problems in identity development in general and in the vocational domain specifically) may affect psychological wellbeing in a negative way, and that intervention that stimulates the development of vocational identity reduces psychological problems as well.

## OBJECTIVES OF THE PRESENT STUDY

The main objective in the present study is to investigate whether the level of psychological problems of the participants decreases following a career choice intervention. To start with, we controlled whether – in line with the research findings discussed above – also in our sample the participants of the career choice intervention had above average levels of psychological problems before they started with the intervention. Hypothesis 1: the participants report above average levels of different types of psychological problems before the start of the intervention.

To get insight in the effect of a career choice intervention on psychological problems we investigated the effect of the career choice intervention on the score for psychological problems. Based on identity theory, we started from the assumption that problems in identity development precede the psychological problems and thus, that a career choice intervention would reduce participants’ psychological problems. Hypothesis 2: the average level of psychological problems decreases after the intervention.

In addition, we investigated the differential effect of the career choice intervention for participants with different levels of psychological problems before the start of the intervention. We expect that the intervention will be equally effective in participants with low to average and with and (very) high levels of psychological problems. Hypothesis 3: there is no significant difference in the effect of the intervention on vocational, personal, and global commitment development for participants with below or average levels of psychological problems, and participants with (very) high levels of psychological problems.

Finally we explored the reduction of the level of psychological problems is also found in the group with (very) high initial levels of psychological problems.

## MATERIALS AND METHODS

### PARTICIPANTS

All participants in our study are participants of the Career Intervention Program (Saxion Orientatieproject). They enrolled in the program because they wanted help for their career choice problems. All 120 participants of this program in 2009, 2010, and 2011 were invited to participate in the study. Half of them agreed and participated in the pre-project interviews. Of these 60 participants, 25% either dropped out during the program or did not participate in the second interview session after the program. The most common reason for attrition was that the subjects were too busy, ill, or on holidays at the time of the second interview. The 45 participants that participated in both interviews did not differ from the 15 dropouts with regard to the number of psychological problems, identity scores, or with regard to demographic variables as age and gender. All participants were Dutch citizens. The mean age is 19.3, see **Table 1** for the distribution of the ages.

**Table 1:** Age of participants in half year groups.

Age group in years and month	Frequency
17.0–17.5	4
5 17.6–17.11	3
18.0–18.5	9
18.6–18.11	8
19.0–19.5	7
19.6–19.11	9
20.0–20.5	2
20.6–20.11	2
21.0–21.5	4
21.6–21.11	4
23.0–23.5	1
23.6–23.11	1
24.0–24.5	1

Twenty four (53%) of the participants are women. All participants have finished the middle or highest level of the Dutch secondary school. At age 12, after finishing primary school, adolescents in the Netherlands choose one out of three levels of secondary education. These different levels give entrance to different types of tertiary education. The middle educational level, called Havo, lasts 5 years and offers entrance to higher levels of professional training and universities for applied sciences. The highest secondary school level, called VWO, lasts 6 years. This level gives entrance to all universities. About half the participants have recently finished their secondary school; the other half has started a study for at least 1 year and dropped out. The participants apply for the project because they experience problems in making a career and/or educational choice and seek for help. They do not follow a study at the time of the program.

### MEASUREMENTS

To assess the level of vocational, personal, and global commitment we administered an identity interview and a symptom checklist before and after the project. The identity interview, the Groningen Identity Development Scale (GIDS; Bosma, 1985) is a semi-structured interview that measures the level of exploration and of commitment strength and the commitment content in different domains. It focuses on six specific domains (philosophy of life, parents, friendship, vocational domain, personal domain, and intimate relationships) and on global identity. In this study we use the results of the target domains of career choice intervention: the vocational and personal domain and global identity. The interview is administered by master students in developmental psychology, who were trained in administering the GIDS interview. For each domain, the method starts with an open interview in which the participants are stimulated to tell about themselves with regard to this domain. Next, the participants are asked to write on a card what is most important for them with regard to that particular domain. This statement is the commitment. As a next step, a questionnaire is administered about the statement on the card. This questionnaire consists of a C-scale (18 items) for commitment strength [example: “do you feel involved in.. (this commitment)..?”] and an E-scale (14 items) for exploration [example: “Do you talk with others about this … (topic)?”]. Each item can be answered with “often/a lot” (score 2), “more or less” (score 1), and “almost never” (score 0). In this way a sum score is obtained per domain for exploration (range 0–28), and for commitment strength (range 0–36). Following the interviews about all domains, the participant is asked to formulate a domain overarching commitment: the global identity. For this, the interviewer places all six cards of the different domains in front of the participant, and asks whether there is something general in these different cards, something that is common, overarching. Different descriptions are used to make clear what is meant. After the participant writes down the general statement, a questionnaire is administered in the same way as for the domain specific cards. The second identity interview was administered in the same way as the first, except that in the open interview questions were added about changes during the previous 6 months. Psychometric analysis of the GIDS (Kunnen, 2013b) demonstrated that the construct validity and reliability were satisfying. The commitment scales in all domains had a reliability (alpha) score above 0.80. The alpha of the exploration scale in the vocational domain was 0.65, the exploration scales of all other domains had an alpha above 0.70 (Kunnen, 2013b).

The level of psychological problems was measured by means of the Dutch version of the Symptom Check List SCL-90 (Arindell and Ettema, 1986), a standard checklist for a broad range of psychological problems. The 90 items result in scores for anxiety, agoraphobia, somatization, depression, sleeping problems, obsessive compulsive problems, suspiciousness, interpersonal sensitivity, hostility, and psycho neuroticism (total score). In the literature, a broad range of psychological problems are addressed such as for example low levels of self-esteem and adaptation, and higher levels of anxiety and depression. For that reason we decided not to focus on one type of problem but to use a screening method that assesses a wide range of psychological problems. We use the total score, representing a general level of psychological problems, but in addition we explore the level of the specific types of problems. To assess whether our participants score below or above average, we compared the results in our study to the norm group that consists of a representative sample (*n* = 2394) of Dutch inhabitants. The gender distribution in our sample and in the norm group is equal. The mean age of our group is significantly lower (41 against 19 year). However, there are no systematic significant relations between SCL-scores and age. Only for hostility and sleeping problems younger people tend to have higher scores, but for both scales, the relation with age was too low and inconsistent to have implications for the test norms. The norm scores for the total scale run from 1 to 7 in which 4 is average, 5% of the population scores 1, 15% scores 2, 15% scores 3, 30% scores 4, 15% scores 5, 15% scores 6, 5% scores 7. The other scales have different norms. For one subscale (agoraphobia) the norm scores run from 1 to 3 in which 2 is the average level, for the scales anxiety, insufficiency of thinking and acting, hostility and sleeping problems the norm scores run from 1 to 4 in which 2 is the average level, the depression scale runs from 1 to 5 in which 3 is the average level, and the scales for somatization and for obsessive compulsive problems run from 1 to 6 in which 3 is the average level. The reliability (alpha) runs from 0.76 for the hostility scale to 0.97 for the total scale.

### INTERVENTION

The career choice intervention we used in our study is the Saxion Orientation Project, part of the Saxion University of Applied Sciences in Deventer, the Netherlands (Saxion Orientatieproject, 2012). Since 20 years this Saxion career guidance project offers a 4 month guidance program for young people with career choice problems. Although the project is part of the Saxion University, it is explicitly meant for participants who are not students of the university (or another school or university) at this moment, because for students other help is available (for free). The participants have to pay a fee of about 1000 €. The intervention is explicitly designed to stimulate career choice and identity development. It stimulates both exploration skills, and decision making skills. The aim of this program is not only to help the participants to make a choice, but also to help to explore themselves, and to develop ideas about what is really important to them, who they are, and who they want to be. In other words, it aims to stimulate identity development. Previous studies showed that the project is effective, firstly because it has a positive effect on commitment development (Kunnen, 2013a), and secondly, because almost all participants made a career choice following the project and 85% (against 66% in general) of the participants has kept to that choice for at least 1 year. The other 15% stated that they are better able to make new choices than they were before (Saxion Orientatieproject, 2012). The program takes 2 days per week, and consists of group meetings and individual sessions, and homework assignments. Each group consists of 10–12 participants, and is supervised by one experienced and certified career choice counselor. The assignments are firstly directed toward exploring oneself, one’s strong and weak points, interests, values etc. These assignments have the form of written assignments, of group exercises and presentations. Secondly the assignments are directed toward exploring the world of studies and work: by visiting companies, schools and universities, and gathering information about different possible studies. Finally the assignments concern the ordering and selecting of the available information, they offer strategies about how to make selections and determine preferences, and finally to make a choice. In the group meetings there is exchange of the results of the assignments and the experiences of the participants. The assignments and meetings concern both cognitive informational aspects, and personal emotional aspects. The different assignments in the guidance resemble five of the six steps as formulated by Germeijs and Verschueren (2007): (2) self-exploration, (3) broad exploration of the environment, (4) in-depth exploration of the environment, (5) decisional status, and (6) commitment. Although there is time to discuss personal problems such as fear of failure during one or two private sessions with the counselor, the focus is not on the treatment of psychological problems, but on the development of (vocational) identity. A more detailed description of the intervention program is available at request from the author of this paper.

### ANALYSIS

To test hypothesis 1, whether the initial level of psychological problems (thus the SCL-90 outcomes on the different subscales and the global score) was above average, we performed a one sample *t*-test with the average scores as given in the manual as criterion. To test hypothesis 2, we compared the levels of specific and the overall psychological problems before and after the intervention by means of a paired sample *t*-test. In addition, we compared the participants’ levels of psychological problems after the intervention with the norms of a standard population by means of a one sample *t*-test, in which we entered the average population score as criterion. Because these changes concern the effects of an intervention, for the domains that showed a significant change also computed the effect size by means of Cohen’s d (the difference between both measurements divided by the average standard deviation of both measurements).

To test hypothesis 3 we compared by means of an independent sample *t*-test the change in the commitment development of the participants who initially scored (below) average on the total scale of psychological problems (score 1–4) with the participants who scored high or very high (score 6–7). In the same way we analyzed the reduction in the level of psychological problems in both groups.

## RESULTS

Our first step was to control whether there were significant relations between the initial levels of the psychological problems, and the variables gender and age. No significant differences were found between men and women. With regard to age, only the sub scale obsessive compulsive complaints showed a significant relation (*r* = 0.29, *p* < 0.05) with age, indicating that the older participants tended to have a higher score on this scale.

Secondly, we tested whether also in our sample the average levels of psychological problems were above average. Before the start of the intervention, the total level of psychological problems level was significantly higher in the group of participants as compared to the norm group. With regard to the sub scales the participants reported significantly higher levels of depression, obsessive-compulsive problems, interpersonal sensitivity, and hostility. No significant difference was found for the sub scales phobia, anxiety, sleeping problems, and somatization. This is shown in the first two columns of **Table 2**. This confirms hypothesis 1.

**Table 2:** Norm scores of the SCL-90 (psychological problems list) before and after the intervention, compared to the average of the norm group (*n* = 45).

	Average of norm group	Mean before	*T*-score before	Mean after	*T*-score after
Anxiety	2	2.00	0.00	1.67**	-2.476
Phobia	1.5	1.45	-0.574	1.40	-0.829
Depression	2	2.65***	3.565	2.20***	0.964
Somatization	3	3.12	0.633	2.58**	-2.062
Obsessive-compulsive	2	2.65***	5.724	2.02	0.167
Interpersonal sensitivity	3	3.52**	2.502	3.24	1.017
Hostility	2	2.28**	2.429	1.82	-1.345
Sleeping problems	2	2.17	2.17	1.93	-0.453
Total	4	4.62***	3.099	4.0	0.00

Difference between mean score and norm score **p < 0.05; ***p < 0.01.

Following the career choice intervention the total level of problems was significantly lower than it was before the start of the intervention. Also the levels of anxiety, depression, somatization, obsessive-compulsive problems, interpersonal sensitivity, and hostility decreased significantly following the intervention (**Table 3**). Only for phobia and sleeping problems no significant decrease was found. The effect sizes are small (anxiety, depression, somatization, and interpersonal sensitivity) to medium (hostility, obsessive-compulsive, and the total level of psychological problems). After the intervention the participants reported less anxiety and somatization as compared to the norm group. For the other problems and the total score they reported levels comparable to the norm group (**Table 2**). These findings confirm hypothesis 2.

**Table 3:** Raw scores of the SCL-90 (psychological problems list) before and after the intervention (*n* = 45).

	Mean T1	SD T1	Mean T2	SD T2	*T*-score	*P*-value	Effect Size	% improve	% worse
Anxiety	13.7	4.11	12.6	3.40	1.821	0.038**	0.29	53	22
Phobia	8.3	1.77	8.2	2.34	0.412	0.342	0.31	55	29
Depression	25.0	8.90	22.4	7.88	1.992	0.027**	0.29	60	29
Somatization	17.0	4.88	15.6	4.65	2.464	0.009***	0.42	69	20
Obsessive-compulsive	15.8	5.33	13.6	5.21	2.993	0.003***	0.31	62	27
Interpersonal sensitive	28.1	9.01	25.5	7.88	2.273	0.014**	0.48	60	13
Hostility	8.4	2.20	7.4	1.98	3.717	0.003***			
Sleeping problems	4.9	2.17	4.6	2.14	1.006	0.160			
Total	133.4	33.42	121.6	32.72	2.703	0.005***	0.36	78	20

**p < 0.05; ***p < 0.01.

We analyzed whether the effect of the intervention differs between participants with low or average levels of psychological problems, and participants with (very) high levels of psychological problems.

With regard to the change in commitment development no significant differences were found between participants who started the intervention with below or above average levels of psychological problems (**Table 4**). The vocational, personal, and the global commitment increased in strength in both groups. There were no systematic differences between the groups, and the difference between the groups was not significant. Thus, hypothesis 3 is confirmed. The level of psychological problems decreased only in the group of participants that initially had (very) high levels of problems.

**Table 4:** Mean changes in commitment score in the target domains and in psychological problems for participants with low or average and with (very) high levels of psychological problems before the start of the intervention.

		Commitment change in			Change in
Initial level of psychological problems	*N*	Vocational identity	Personal identity	Global identity	Psychological problems
Low to average	23	4.1	3.5	3.2	0
(very) High	19	2.6	3.6	5.8	-1.1
Significance (2-sided)		0.48	0.96	0.28	0.02

*Level of problems : low to average, score 1, 2, 3, 4; high and very high score 6, 7.*

## DISCUSSION

Our findings confirmed the first hypothesis, stating that the participants of the career choice intervention had above average levels of psychological problems before the start of the intervention. The participants have significantly above average scores for the total level of problems. With regard to the sub scales the difference was significant of 4 out of 8 subscales: Depression, obsessive-compulsive, interpersonal sensitivity, and hostility problems. With regard to hostility it should be kept in mind that the relative young age of the participants, as compared to the norm group, may contribute to the difference because this score is negatively related to age. However, that is not the case for the other scales. We come back to the different types of problems later. These findings are in line with findings of for example Creed and Patton (2003), Creed et al. (2004), Kunnen et al. (2009), and Rottinghaus et al. (2009) who also found a higher level of psychological problems in late adolescents with career choice problems.

In line with hypothesis 2 the level of psychological problems was found to decrease significantly following the career choice intervention. These findings are in line with the research of Skorikov (2007) and Skorikov and Vondracek (2007) who also find that psychological problems reduced following a career preparation. The levels of psychological problems following the intervention were around average.

Also hypothesis 3 is confirmed: the initial level of psychological problems is not significantly related to the effects of the intervention on commitment development: the intervention is equally effective in participants with low or average and with (very) high levels of psychological problems. These findings suggest that high levels of psychological problems do not necessarily form a contra indication for career choice intervention. On the contrary, concerning the career choice development, the intervention is equally effective for participants with (very) high levels of psychological problems as for other participants but in addition, the level of psychological problems reduced significantly in this group. The differences in reduction between both groups should be considered with caution, because bottom effects may have played a role in the group of participants who already started with low or average levels of psychological problems. The results are meaningful however, in that they show that the high levels of psychological problems do decrease especially in the group with initially very high levels of problems following the intervention.

This means that the answer on the question of career choice counselors can be that psychological problems need not be a contra indication for career choice intervention. This does not mean of course that all psychological problems can be solved by career choice intervention. Firstly the participants in this study themselves made the choice for a career choice intervention, so, they perceived their career choice problems as the most urgent ones. Secondly, this study does not address severe psychopathology or people with diagnoses such as anxiety disorders or depression. The measurement of psychological problems in this study concerned self-reports of feelings of anxiety, depression etc.

Our findings lend some support for the assumption of Montgomery et al. (2008) that at least for our participants identity problems may be at the core of other problems, in this case psychological problems. This does not necessarily mean that the psychological problems were caused by the identity problems. In this paper we deliberately avoided to suggest direct causal relations between psychological problems and identity problems. Most probably, these different problems interact with each other. Identity can be seen as a complex dynamic system (Kunnen, 2011) and as such, identity and its interacting factors do not form simple causal relations, but form a complex system in which they may hold each other in place. Interventions that address a part of the system that is highly relevant at that moment may cause desequilibrium and a reorganization of the system. In this paper we analyzed a broad range of psychological problems, and considered them as more or less additive, because our target dependent variable was the total score. We did not formulate specific expectations for different types of problems. This is partly because the literature does address a broad range of psychological problems, ranging from low self-esteem and efficacy, to depression and non-effective coping strategies, and from research no clear picture emerges with regard to the relation between career choice and different types of psychological problems. But also from a theoretical point of view we think that it is better to start with a more global analysis of the relation between identity problems and psychological problems. Because we see both types of problems as part of one complex system, we assume that it will be difficult to distinguish between relations of different specific psychological problems. However, to analyze whether specific types of problems play different roles in this system remains a challenge for later research.

A complicating factor in the interpretation of studies into the effects of career choice counseling research on psychological problems is the large variation in target groups, in types of intervention and in the organization of interventions in different countries. For example, the above mentioned studies of Skorikov (2007) that demonstrated a positive effect of career choice counseling on psychological problems concerned a general prevention program. These studies focused on the longitudinal analysis of the development of broad groups of high school students, and as such, their findings concern the relations between career choice and adjustment and wellbeing in the broad population. This is most probably another population than the specific population of late adolescents who seek help for their career choice problems by enrolling in an intervention. The career choice intervention in this study is of the latter type. Career choice guidance during secondary school in the Netherlands consists of general guidance that is offered to all students, although the content and form differs greatly between schools. However, career choice help for late adolescents who have finished secondary school or who have dropped out from tertiary education is not supported by the health care services, and thus the participants have to pay for these interventions. In the Netherlands this help is offered by individual career choice counselors who charge a fee per hour or by intensive intervention programs. These programs take between 4 months to 1 year, are half time or full time, and cost between 1000 and more than 6000 €. The program in our study takes 2 days for a period of 4 months, and costs about 1000 €. The fact that adolescents in such programs actively seek help suggests that – as compared to the average population – their problems may be more severe and experienced as urgent, or maybe, that these students cope with their problems in an more active way. Probably, late adolescents who attend career choice intervention programs form a selective subgroup in which the dynamics between career choice problems, well-being and psychological problems differ from those in the average population. Knowledge about the dynamic relations in this group between career choice problems, psychological problems, and the effects of career choice counseling on these problems is especially relevant for practitioners who have to decide which problems should be addressed first, and whether they accept participants with serious psychological problems in their intervention. At this moment, it means that comparison of the outcomes of different career choice intervention studies should be done with great caution, and with attention for the possible differences between the types of intervention and the target groups.

Several questions are left unanswered in this study. Firstly, although we have evidence that the career choice intervention at the same time stimulates development in the relevant identity domains and decreases the level of psychological problems, we still have no insight in the micro level processes: what happens exactly during the intervention? Is there a direct effect of increase in commitment strength on the psychological problems? Or do the problems decrease already during the project, as a consequence of the participation itself? Are there specific parts that trigger development? What inter-individual differences exist? Is amount of effect on psychological problems related to the effect on commitment strength? Are there different patterns of change? This kind of questions can be answered only by micro level process research, in which the participants are assessed frequently.

In addition, a limitation in this study is that we assessed psychological problems by means of a self-report instrument. Although this is the most common used instrument in the Netherlands both in research and in the clinical practice, it would contribute to our knowledge to use different instruments to assess psychological problems, and to add observations, or reports from others.

Another limitation is the fact that this study concerns only one type of career intervention, and due to the rather high fees, a group of participants who were motivated and who could afford to pay this fee. For firm and general conclusions, we have to study different types of intervention and different groups of participants.

However, despite the small number of participants in this study, the main results are highly significant: even participants who entered the intervention with (very) high levels of psychological problems gained from the intervention both in terms of identity development and in terms of reduction of psychological problems. These findings contribute to our understanding of the dynamic interaction between career choice problems and psychological problems. Although more research is needed, our outcomes suggest an answer to the practitioners’ questions that was the first reason for our study: psychological problems as indicated by high scores on self-report measures are not necessarily a contra indication for career choice intervention. Instead, solving the career and identity problems may result in a reduction of the psychological problems.

## Conflict of Interest Statement

The author declares that the research was conducted in the absence of any commercial or financial relationships that could be construed as a potential conflict of interest.
